# Is nano safe in foods? Establishing the factors impacting the gastrointestinal fate and toxicity of organic and inorganic food-grade nanoparticles

**DOI:** 10.1038/s41538-017-0005-1

**Published:** 2017-11-20

**Authors:** David Julian McClements, Hang Xiao

**Affiliations:** Department of Food Science, University of Massachusetts Amherst, Amherst, MA 01003 USA

**Keywords:** Cell-particle interactions, Nanoparticles

## Abstract

Nanotechnology offers the food industry a number of new approaches for improving the quality, shelf life, safety, and healthiness of foods. Nevertheless, there is concern from consumers, regulatory agencies, and the food industry about potential adverse effects (toxicity) associated with the application of nanotechnology in foods. In particular, there is concern about the direct incorporation of engineered nanoparticles into foods, such as those used as delivery systems for colors, flavors, preservatives, nutrients, and nutraceuticals, or those used to modify the optical, rheological, or flow properties of foods or food packaging. This review article summarizes the application of both inorganic (silver, iron oxide, titanium dioxide, silicon dioxide, and zinc oxide) and organic (lipid, protein, and carbohydrate) nanoparticles in foods, highlights the most important nanoparticle characteristics that influence their behavior, discusses the importance of food matrix and gastrointestinal tract effects on nanoparticle properties, emphasizes potential toxicity mechanisms of different food-grade nanoparticles, and stresses important areas where research is still needed. The authors note that nanoparticles are already present in many natural and processed foods, and that new kinds of nanoparticles may be utilized as functional ingredients by the food industry in the future. Many of these nanoparticles are unlikely to have adverse affects on human health, but there is evidence that some of them could have harmful effects and that future studies are required.

## Introduction

Nanotechnology involves the development, characterization, and application of materials with length scales in the nanometer range (typically 1–100 nm).^1^ Controlling the structure and properties of materials at this length scale can lead to novel properties that are beneficial for certain commercial applications. In the food industry, nanotechnology can be utilized to improve food quality, shelf life, safety, cost, and nutritional benefits.^2^ In some cases, the nanomaterials used in the food industry are not intended to find their way into the final food product, e.g., those used in packaging, sensors, and antimicrobial treatments designed for sanitizing food manufacturing plants. In other cases, nanomaterials are specifically designed to be incorporated into food products, such as nanoparticles used as delivery systems or to modify optical, rheological, or flow properties. This review article focuses on the properties and potential safety of ingested nanomaterials, since they are most likely to cause health concerns. It is important to distinguish different potential sources of nanoscale materials found in foods. Nanoscale materials are naturally present in many commonly consumed foods, such as the casein micelles in milk or certain organelles found in plant or animal cells.^3–5^ Engineered nanoscale materials (ENMs) may be intentionally added to foods (such as nanoparticle-based delivery systems), or they may inadvertently find their way into foods (such as nanoparticles in packaging materials that leach into the food matrix).^6–8^ ENMs are typically nanoparticles whose composition, size, shape, and interfacial properties are specifically designed to achieve one or more functional attributes. In particular, ENMs may be used to create delivery systems for nutrients, nutraceuticals, colors, flavors, and preservatives, or they may be used to modify the texture, appearance, or stability of foods. Finally, nanoscale structures may be present in foods as the result of routinely used food processing operations, such as homogenization, grinding, and cooking.^9,10^ In this case, the food manufacturer may not be intentionally trying to create nanoparticles, but they are a natural consequence of the processing operations used. Different types of nanoscale materials that may be found in foods, and their potential origins are highlighted in Table 1.

**Table 1:** Examples of different kinds of nanoscale materials that might be present within foods and their origin

Nanoscale material	Origin	Characteristics	Products
*Organic nanoparticles*			
Casein micelles	Natural	Protein–mineral clusters	Milk, cream
Cell organelles	Natural	Ribosomes, vacuoles, lysosome etc.	Meat, fish, fruits, vegetables, spices
Oil bodies	Natural	Phospholipid/protein-coated triglyceride droplets	Plants, seeds
Lipid nanoparticles	ENP	Solid particles or liquid droplets coated by emulsifiers	Some beverages, sauces, dressings, creams
Protein nanoparticles	ENP	Clusters of protein molecules held together by physical or covalent interactions	Mainly in development
Carbohydrate nanoparticles	ENP	Small solid fragments extracted from starch, cellulose, or chitosan. Clusters of polysaccharide molecules held together by physical or covalent interactions.	Mainly in development
*Inorganic nanoparticles*			
Iron oxide	ENP	FeO nanoparticles used to fortify foods with iron.	Nutritional supplements, sausage casings
Titanium dioxide	ENP	TiO_2_ nanoparticles used as whitening agents	Candies, chewing gums, bakery goods, milk powders.
Silicon dioxide	ENP	SiO_2_ nanoparticles used to control powder flowability	*S*alts, icing sugar, spices, dried milk, and dry mixes
Silver	ENP	Ag nanoparticles used as antimicrobials in foods, coatings and packaging	Meat, food packages, containers, coatings

Key: ENP engineered nanoparticle, which may be intentionally or unintentionally added

Potentially, nanoparticles may exhibit either acute or chronic toxicity, but the latter type is the most important in foods since relatively low levels of nanoparticles are likely to be consumed over an extended period. In general, the toxicity of ingested nanoparticles depends on their ability to damage cells or organs within humans, thereby adversely affecting human health or wellbeing.^11^ Cellular or organ damage can occur in various places within the gastrointestinal tract (GIT), as well as after absorption of the nanoparticles into the body.^11^ Moreover, nanoparticles may damage the microbial cells that normally populate the human GIT, which could indirectly alter human health.^12^

## Types of nanoparticles in foods

In general, the nanoparticles present in foods can be conveniently categorized according to their composition, as either organic or inorganic, since this factor has a major impact on their gastrointestinal fate and potential toxicity.

### Inorganic nanoparticles

Many types of nanoparticles used in foods are mainly composed of inorganic materials, such as silver, iron oxide, titanium dioxide, silicon dioxide, or zinc oxide.^13^ These particles are either crystalline or amorphous solids at ambient temperature, which may be spherical or non-spherical, have different surface characteristics, and come in different sizes depending on the initial materials and preparation conditions used in their fabrication. Inorganic nanoparticles also vary in their tendency to dissolve under different solution conditions (such as pH and ionic strength) and in their chemical reactivities, which has a major impact on their GIT fate and toxicity.

#### Silver nanoparticles

Silver (Ag) nanoparticles are used in a variety of applications within the food industry. They have been used as antimicrobial agents in foods and food packaging materials.^14–16^ For example, manufacturers have claimed that silver nanoparticles are used for their antimicrobial effects in certain types of food containers in the US (e.g., Kinetic Go Green basic nanosilver food storage container, Oso fresh food storage container, and FresherLonger^TM^ Plastic Storage bags).^17^ It is possible that some of these silver nanoparticles may migrate from these containers and into foods so that they could be ingested by humans.^17–19^ Silver nanoparticles may also form spontaneously within biological media (such as GIT fluids or foods) when soluble silver salts interact with other components present.^20^ It has been estimated that adults may consume between 20 and 80 μg/day of silver, with only a fraction of this being in the form of nanoparticles.^12^ At present there is still limited information about the potential toxicity of silver nanoparticles ingested with foods,^15^ with some studies reporting no toxicity and others reporting appreciable toxicity. The GIT may be particularly susceptible to silver nanoparticle-induced toxicity since it contains the first tissues exposed to dietary nanoparticles after ingestion. However, the adverse effects of silver nanoparticles on the GIT remain inconclusive. Several animal studies have reported that dietary intake of silver nanoparticles caused lymphocyte infiltration, pigmentation of villi, discharge of mucus granules, and an abnormal mucus composition in the intestine.^21–24^ Animal studies have reported that silver nanoparticles can accumulate in various organs after ingestion, including the liver, kidneys, spleen, stomach, and small intestine.^15,25,26^ These results suggest that silver nanoparticles can be absorbed by the GIT into the systemic circulation, and then be distributed throughout various organs. However, only a small fraction (<1%) of ingested silver nanoparticles typically accumulate in tissues, which suggests that the majority of them were excreted in the feces or urine.^25^ At the levels used in this study (2000 and 250 mg/kg body weight for single and multiple doses, respectively), no toxicity of the silver nanoparticles was found after oral gavage.^25^ Another rat feeding study reported no major toxic effects of ingestion of silver nanoparticles over a 28-day period (30, 300 and 1000 mg/kg day), but that there was some slight liver damage at the highest levels used.^26^ Other studies have also reported some adverse impacts on liver and kidney function of mice upon repeated ingestion of silver nanoparticles.^27,28^ It was suggested that 125 mg/kg of silver nanoparticles may be the limit above which adverse effects on liver may be observed.^28^ In summary, animal studies have shown that silver nanoparticles may accumulate in the body and have toxic effects when ingested at sufficiently high levels, but it is not clear whether these levels are close to those actually achievable through food consumption. In future studies, it will therefore be important to carry out long-term chronic toxicity studies using nanoparticle levels that are more similar to those actually consumed in the human diet. In addition, further studies are required to determine if silver nanoparticles dissolve in gastrointestinal fluids, and to assess whether there is a difference in behavior of silver when ingested in a soluble or nanoparticle form.^29^ Indeed, a study in which rats were fed either soluble or nanoparticle forms of silver found that the organ distribution of the silver was similar in both cases.

Numerous studies using cell culture models have reported that silver nanoparticles may promote cytotoxicity through various mechanisms.^30,31^ Cell culture studies have also shown that the effect of silver nanoparticles depends on their size, with smaller ones being more cytotoxic than larger ones,^32^ as well as on the nature of the coating on their surfaces.^33^ An important factor contributing to the toxicity of silver nanoparticles is their ability to generate reactive oxygen species (ROS) thereby promoting oxidative stress, which results in damage to cell membranes, organelles, and the nucleus.^15,33^ In addition, they may disrupt normal biochemical functions, such as ATP production, DNA replication, and gene expression.^33^ The strong antimicrobial activity of silver nanoparticles may also alter the nature of the gut microbiota, especially if they reach the colon.^12,34^ The effects of the food matrix on the behavior of silver nanoparticles are ignored in cell culture studies, but one recent study showed that certain food components did have an appreciable impact on the absorption and toxicity of silver nanoparticles in intestinal cells.^35^ A common issue with cell culture studies is that the dose of nanoparticles used is much higher than would ever be found in practice, and is therefore not physiologically relevant.

#### Zinc oxide

Zinc oxide (ZnO) nanoparticles may be used as a source of zinc in supplements and functional foods, since this is an essential trace element needed to maintain human health and wellbeing.^36^ ZnO nanoparticles may also be utilized in food packaging as antimicrobial agents to prevent contamination of foods with harmful bacteria^37^ or as ultraviolet (UV) light absorbers to protect foods that are sensitive to UV light exposure.^38^ In principle, nanoparticles in packaging may leach into food products and therefore be ingested as part of the human diet.^39^ However, a recent risk assessment suggests that this does not occur to an appreciable level for ZnO nanoparticles.^38^ The antimicrobial activity of ZnO nanoparticles has been partly attributed to their ability to penetrate into microbial cells and generate ROS that damage key cellular components thereby leading to cytotoxicity.^37^ This mechanism could lead to adverse health effects in humans if this type of nanoparticle were ingested in sufficient quantities and then absorbed by the human body. Several rodent feeding studies have demonstrated particle size-dependent effects on the intestinal uptake of ZnO nanoparticles, with a smaller particle size leading to a higher uptake.^40–43^ One study reported that a single oral dose of ZnO nanoparticles caused hepatic injury, kidney toxicity, and lung damage.^44^ Interestingly, one study showed that ZnO nanoparticles were not toxic when used in isolation, but that they become toxic when mixed with ascorbic acid.^36^ This suggests that it is important to measure the impact of specific food components on the toxicity of this type of nanoparticle.

ZnO nanoparticles may be spherical or non-spherical solid particles that are usually highly aggregated when dispersed in aqueous solutions.^36,45^ These aggregates are typically many times larger than the individual nanoparticles, with their size and structure depending on solution conditions, which is likely to have a major effect on their GIT fate and toxicity. Feeding studies with frogs have shown that zinc oxide nanoparticles exhibit greater toxicity than a dissolved form of zinc, which was attributed to their greater capacity to induce oxidative damage in cells.^46^ This study highlights the importance of establishing the physical form of zinc when ZnO nanoparticles are ingested.

#### Iron oxide nanoparticles

Iron oxide (Fe_2_O_3_) nanoparticles may be utilized in foods as colorants or sources of bioavailable iron.^47–50^ The range of applications of iron oxide as a food colorant in the United States are highly limited, i.e., up to 0.1 wt% in sausages as part of casings.^51^ It was estimated that the mean intake of iron oxide from consumers of these products was around 450 μg/day.^51^ However, the levels of iron consumed may be considerably higher for consumers who take mineral-fortified supplements or functional foods.^52^ For example, iron taken in the form of enriched/fortified foods ranges from 10 to 23 mg/day, while that from dietary supplements may range from 10 to 32 mg/day.^52^ It should be stressed that this type of iron is not usually delivered in the form of iron oxide or as nanoparticles. However, if iron oxide nanoparticles were used for this purpose, then they could be present at these levels. Iron oxide nanoparticles may come in different sizes, shapes, and crystalline forms, which may alter their toxicity.^53^ It has been proposed that the ability of iron oxide nanoparticles to generate ROS is the most likely mechanism for their potential toxicity.^49^ A study where iron oxide nanoparticles were orally administered at about 3 mg/kg body weight to rats over a 13-week period reported that they did not accumulate in tissues or produce toxicity.^54^ Another rat feeding study with much higher oral dose (250–1000 mg/kg body weight) of iron oxide nanoparticles for 13-week also did not find their tissue accumulation or toxicity in both male or female rats.^50^

#### Titanium dioxide (TiO_2_) nanoparticles

**Fig. 1:**
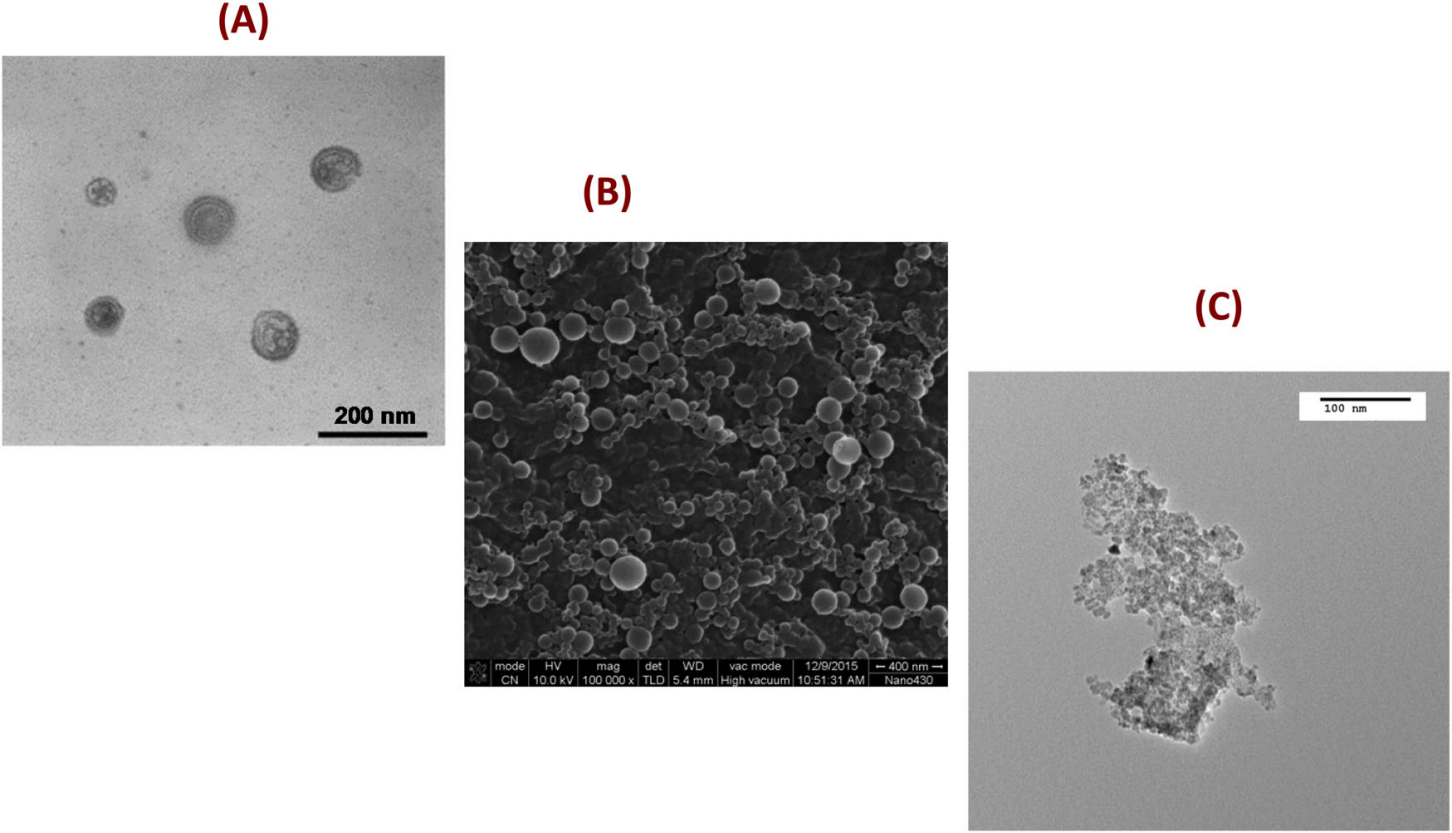
Some examples of different kinds of organic and inorganic nanoparticles that may be present within foods, such as: **a** lipid nanoparticles (protein-coated oil droplets); **b** protein nanoparticles (polysaccharide-coated zein particles); **c** Titanium dioxide nanoparticles (all images taken in our laboratory)

TiO_2_ particles are used as functional ingredients in certain foods to provide characteristic optical properties such as increased lightness and brightness.^55^ Typically, the TiO_2_ ingredients utilized in the food industry as lightening agents are optimized to have particle sizes of 100–300 nanometers to increase their light scattering properties.^56^ Nevertheless, these ingredients contain a range of different particle sizes and there may be a significant proportion of particles with diameters <100 nm, which can therefore be considered to be nanoparticles.^57^ For example, the mean diameter of the particles in food grade TiO_2_ (E171) powders obtained from several manufacturers was reported to be about 110 nm, with >36% of the particles being below 100 nm.^58^ The estimated dietary exposure of humans to TiO_2_ nanoparticles has been reported to be up to 1.1 and 2.2 mg/kg body weight/day in the UK and US, respectively.^58^ Chewing one piece of chewing gum can result in an intake of 1.5–5.1 mg of TiO_2_ nanoparticles.^59^ It is noteworthy that the amount of TiO_2_ nanoparticles consumed was 2–4 times higher for children than for adults, which may be due to the fact that products heavily consumed by children had some of the highest levels of TiO_2_ nanoparticles, such as candies, gums, desserts, and beverages. TiO_2_ nanoparticles may vary in their sizes, shapes, crystal form, interfacial properties, and aggregation states, which will impact their GIT fate and toxicity. A representative scanning electron microscopy image of titanium dioxide particles is shown in Fig. 1. The most common crystalline forms in food-grade titanium dioxide particles are anatase and rutile, which are polymorphic forms that have different crystal packing and physicochemical properties.^60^ The surface composition of food-grade titanium dioxide particles may also vary depending on the source, with different levels of phosphorous, aluminum and silica being detected by X-ray analysis.^60^ In addition, organic molecules may also be present at the particle surfaces, which will also impact their interfacial characteristics, such as ζ-potential, hydrophilicity, surface energy, and chemical reactivity.

A recent review of 16 animal studies concluded that TiO_2_ nanoparticles not only concentrate, accumulate, and magnify in the tissues of mammals and other vertebrates, but that they also have a very limited elimination rate.^56^ Acute and subchronic studies of the oral toxicity of TiO_2_ nanoparticles have been carried out in rodents. A single oral dose of TiO_2_ nanoparticles (25, 80, or 155 nm at 5000 mg/kg body weight) resulted in their accumulation in the liver, spleen, kidney, and lung tissues of mice, and also led to hepatic injury, nephrotoxicity and myocardial damage.^61^ In another study, the anatase form of TiO_2_ nanoparticles (5 nm) was intragastrically administered to mice at 62.5, 125 and 250 mg/kg body weight for 30 days.^62^ At the higher dose, the TiO_2_ nanoparticles caused damages to liver function, the hemostasis blood system, and immune response.^62^ In the intestine, TiO_2_ nanoparticles induced inflammatory cytokine production, T-cell proliferation, hypertrophy, and hyperplasia in the mucosal epithelium.^63,64^ In contrast other studies have reported little accumulation or toxicity of ingested TiO_2_ nanoparticles. For example, a study where TiO_2_ nanoparticles (mixture of anatase and rutile at 21 nm) were repeatedly administered (260–1041 mg/kg) to rats did not report any significant toxicity or TiO_2_ accumulation in tissues or urine, but reported high concentrations of titanium dioxide in feces, suggesting that the TiO_2_ nanoparticles were mostly eliminated.^65^

The observed contradictions between different animal studies on the accumulation and toxicity of TiO_2_ nanoparticles may arise for a number of reasons. Firstly, there are differences in the oral dose, crystal form, particle size, aggregation state, and surface characteristics of the nanoparticles used. Second, the impact of the food matrix and GIT passage on the properties of the nanoparticles are often ignored, or taken into account differently. Third, the type of animal model and analytical methods used to determine accumulation and toxicity may vary. For example, it has been shown that the age of the experimental animals used is an important factor. The same doses (up to 200 mg/kg body weight per day for 30 days) of TiO_2_ nanoparticles (anatase at 75 nm) were used to treat both young (3-week-old) and adult (8-week-old) rats.^66^ Heart injuries, liver edema, and non-allergic mast cell activation in stomach tissue were observed in young animals, but only slight toxic effects were observed in adult animals.^66^ This finding is particularly important given that the amount of TiO_2_ nanoparticles consumed by humans is estimated to be appreciably higher for children than adults.

Numerous cell culture studies have suggested that TiO_2_ nanoparticles may be absorbed by model epithelium cells and produce cytotoxicity depending on their particle characteristics, such as dose, size, crystal form, and surface coating.^67–71^ The ability of TiO_2_ nanoparticles to exhibit these toxic effects has been attributed to numerous mechanisms, including generation of ROS species that damage key cellular constituents, interference with efflux pumps and nutrient transporters, induction of inflammation, and alteration of the gut microbiota.^12,72,73^ Anatase TiO_2_ nanoparticles were reported to be more toxic to cells than rutile nanoparticles due to their higher photo-catalytic activity.^74^ A cell culture study showed that a mixture of anatase and rutile forms caused more severe cytotoxic and genotoxic damage than pure anatase or pure rutile titanium dioxide nanoparticles.^75^ However, this result is in contrast to another animal study where a mixture of anatase and rutile forms of TiO_2_ nanoparticles did not cause significant toxicity in rats.^65^ One possible reason is that cells were exposed to pristine-TiO_2_ nanoparticles in the serum-free media used in the cell culture study, but they were exposed to coated TiO_2_ nanoparticles in the animal studies (because the nanoparticle surfaces adsorb substances from the surrounding GIT fluids). This discrepancy may therefore be due to differences in the biological fate and toxicity of TiO_2_ nanoparticles with different interfacial properties. Indeed, it is well documented that the presence or absence of serum in cell culture media modulates the absorption and toxicity of nanoparticles in cell culture models.^76,77^

#### Silicon dioxide nanoparticles

Silicon dioxide (SiO_2_) nanoparticles are added to certain powdered foods as anticaking agents to enhance flow properties, e.g., salts, icing sugar, spices, dried milk, and dry mixes.^78,79^ Silicon dioxide particles are usually amorphous solid spheres. The majority of particles in food-grade SiO_2_ ingredients (E551) are usually in the 100 to 1000 nm range, but there may also be a significant population of smaller particles. It has been estimated that the intake of SiO_2_ is around 20–50 mg/day per person.^12^ Studies have reported that the individual nanoparticles in commercial SiO_2_ ingredients typically have diameters from about 10 to 50 nm, but that these nanoparticles often exist in the form of larger clusters, usually in the range 100–1000 nm.^80^ Cell culture and animal feeding studies suggest that high levels of SiO_2_ nanoparticles may cause adverse effects, such as cytotoxicity and generation of ROS.^80,81^ A recent study suggested that SiO_2_ nanoparticles accumulate in the liver at levels that could cause a health risk.^82^ Another study showed that feeding of amorphous SiO_2_ nanoparticles to mice for 10 weeks increased the level of ALT (alanine aminotransferase), suggesting a potential adverse effect on the liver.^83^ Conversely, a study that employed oral administration of silicon dioxide nanoparticles to rats over a 13-week period reported no accumulation or toxicity.^54^ Therefore, no clear conclusion on the toxicity of silicon dioxide nanoparticles can be drawn based on the available evidence.

#### General comments

The authors note that many of the review articles published in this area emphasize that there is currently a lack of detailed understanding about the gastrointestinal fate and toxicity of different kinds of inorganic nanoparticles, and that there are often inconsistencies between different studies. There are a number of factors that may contribute to this uncertainty. The types and levels of nanoparticles used in different studies vary considerably, and the levels used in cell culture and animal studies are often much higher than those that would ever be consumed by humans. In addition, simple cell culture models (such as Caco 2 cells) cannot mimic the complexity of animal and human GITs. It should also be noted that the levels of inorganic nanoparticles reported to accumulate in tissues are often misleading, since the analytical techniques used only measure the concentration of specific elements present (such as Ag, Zn, Fe, Ti, or Si) rather than the physical form (e.g., dissolved, nanoparticle, or microparticle). Finally, food matrix effects are often ignored, and may have a pronounced impact on the behavior of nanoparticles in the GIT (see later). It is therefore clear that further systematic research using well-defined nanoparticles and test methods are urgently needed (Section 6).

### Organic nanoparticles

This type of nanoparticle is primarily composed of organic substances, such as lipids, proteins, and/or carbohydrates. These substances tend to be liquid, semi-solid (gelled), or solid (crystalline or amorphous) at ambient temperatures depending on their composition and processing conditions. Most organic nanoparticles commonly used in foods are spherical, but they may be non-spherical under some circumstances (e.g., nanofibers). Organic materials vary considerably in their behaviors within different regions of the human GIT, e.g., they may dissolve, precipitate, aggregate, or be digested in the mouth, stomach, small intestine, or colon depending on their compositions and structures. In general, it is thought that organic nanoparticles are less toxic than inorganic ones, because they are often fully digested within the human GIT and are not bio-persistent. Nevertheless, there may be certain circumstances where they could cause toxicity (Section 5).

#### Lipid nanoparticles

Lipid nanoparticles are widely present within many commercial food products, and are being investigated for their application in other products.^84^ Beverage emulsions, such as soft drinks, fortified waters, fruit juices, and dairy drinks, contain small oil droplets dispersed in water.^85^ An appreciable fraction of the oil droplets in these products falls into the nanoscale range (*d* < 100 nm). Lipid nanoparticles are also being developed as colloidal delivery systems to encapsulate, protect, and release hydrophobic bioactives, such as colors, flavors, antimicrobials, antioxidants, nutrients, and nutraceuticals.^86–89^ The major advantages of using lipid nanoparticles for these applications is that they can increase the bioavailability and/or functional performance of encapsulated components, they can be designed to be optically transparent (which is desirable for clear foods and beverages), and they can increase the physical stability of the product (since small particles are less susceptible to gravitational separation and aggregation).^84,90^ Different types of lipid nanoparticles may be present in foods, including micelles, vesicles, oil droplets, and fat crystals, which vary in their compositions, structures, and dimensions. The diameter of lipid nanoparticles may vary from a few nanometers (surfactant micelles) to a few hundred nanometers (oil droplets or solid lipid nanoparticles). The type of molecules present at their surfaces determines the electrical characteristics of lipid nanoparticles.

Food-grade lipid nanoparticles are usually comprised of either neutral lipids (such as triacylglycerols (TAGs), diacylglycerols (DAGs), monoacylglycerols (MAGs), hydrocarbons, and terpenes) or polar lipids (such as free fatty acids (FFAs), surfactants, and phospholipids).^90^ The GIT fate of lipid nanoparticles depends on their susceptibility to hydrolysis by digestive enzymes, such as lipase and phospholipase.^91^ Many types of lipids are hydrolyzed by lipases in the GIT, including TAGs, DAGs, and phospholipids. These lipids usually have a hydrolysable ester bond that is cleaved to release FFAs and MAGs. FFAs and MAGs can be incorporated into mixed micelles and then be absorbed by the epithelium cells.^91^ Digestible lipid nanoparticles are usually rapidly hydrolyzed in the GIT due to their high specific surface area, which means that they are unlikely to be directly absorbed in their intact state. Consequently, one would not anticipate that this type of nanoparticle would promote toxicity due to absorption and accumulation in intestinal cells and other organs. On the other hand, their ability to increase the bioavailability of hydrophobic bioactive agents may lead to some unforeseen undesirable effects (Section 5).

Certain types of lipid nanoparticles that are currently used in foods, or that may be used in the future, are not digested by the enzymes in the GIT.^90,91^ This may occur because the oil phase itself used is indigestible, such as the terpenes and hydrocarbons found in some flavor, essential, or mineral oils. Alternatively, this may occur because the oil droplets are coated by an interfacial layer that inhibits the digestive enzymes from hydrolyzing the encapsulated lipids.^92^ In these cases, it may be possible for indigestible lipid nanoparticles to be absorbed intact by the human body. Nevertheless, to the authors’ knowledge there have been no studies on the potential fate of this type of nanoparticle after absorption, or of their potential toxicity.

#### Protein nanoparticles

The most common protein nanoparticles found in foods are the casein micelles found in bovine milk and other dairy products, which are small clusters of casein molecules and calcium phosphate ions.^3,4^ As this type of nanoparticle has been widely consumed by humans for many centuries there is little concern about its potential toxicity. Indeed, the nanostructure of casein micelles probably arose through nature’s need to provide an efficient method of delivering nutrients (proteins and minerals) to infants.^93^ Recently, there has been interest in developing other types of protein nanoparticle for application in foods.^86,94,95^ In particular, protein nanoparticles are being developed to create delivery systems to encapsulate, protect, and deliver bioactive agents, such as colors, flavors, preservatives, vitamins, minerals, and nutraceuticals (similar to lipid nanoparticles). Protein nanoparticles usually consist of a cluster of aggregated protein molecules held together by physical interactions (e.g., hydrophobic, hydrogen bonding, van der Waals, or electrostatic attraction) or covalent bonds (e.g., disulfide bonds). Physical bonds are usually formed by altering solution conditions, such as pH, ionic strength, solvent quality, and ingredient interactions, whereas covalent bonds are formed using specific chemical or biochemical reaction conditions. The type of proteins used and the nature of the bonding between them determine the GIT fate of protein nanoparticles. Protein nanoparticles may vary in size from a few nanometers (individual globular proteins) to hundreds of nanometers (e.g., casein micelles, zein, gliadin, whey, and soy protein particles). Typically, protein nanoparticles are spherical, but it is possible to create fibrous structures and other shapes. The electrical charge of protein nanoparticles goes from positive to negative as the pH is increased from below to above the isoelectric point. Protein nanoparticles that are digested in the upper GIT are unlikely to promote toxicity since they produce similar digestion products as conventional forms of proteins (i.e., amino acids and peptides). Nevertheless, the type of peptides produced may be altered, which could alter their allergenicity profile. Indigestible protein nanoparticles could be absorbed by the body or they could interact with the gut microbiota, which could have some unforeseen effects. Protein nanoparticles could also have some effects on human health by altering the bioavailability and/or bioactivities of encapsulated substances, such as minerals, vitamins, or nutraceuticals (Section 5.5).

#### Carbohydrate nanoparticles

Carbohydrate nanoparticles are typically assembled from digestible or indigestible polysaccharides, such as starch, cellulose, alginate, carrageenan, pectin, and xanthan.^96,97^ These nanoparticles can be created by breaking down larger structures found in nature, such as starch granules, chitosan fibrils, or cellulose fibrils. Alternatively, they may be fabricated by promoting the association of polysaccharide molecules, e.g., by changing temperature, utilizing enzymes, or adding specific mineral ions. Carbohydrate nanoparticles may be spherical or non-spherical depending on their origin, and they may be digestible or indigestible within the upper GIT. Certain types of starches are rapidly hydrolyzed by amylases arising from the mouth and small intestine, and thereby converted into oligosaccharides and glucose.^98^ Conversely, other types of starch have structural organizations that make them more or less resistance to hydrolysis by digestive enzymes in the GIT. Most other polysaccharides used to fabricate carbohydrate nanoparticles, collectively known as dietary fibers, are not digested in the upper GIT (mouth, stomach, and small intestine), but may be fermented by enzymes released by the microbiota in the lower GIT (colon). The digestibility of dietary fibers has a major impact on the potential GIT fate of carbohydrate nanoparticles. Carbohydrate nanoparticles that are fully digested in the upper GIT are unlikely to exhibit any toxicity since they produce similar digestion products (simple sugars) as conventional forms of carbohydrates. However, indigestible carbohydrate nanoparticles could be absorbed by the body or interact with the gut microbiota, which could have some adverse health effects. Like protein nanoparticles, carbohydrate nanoparticles could also affect human health by altering the bioavailability and/or bioactivities of encapsulated substances (Section 5.5).

#### Complex nanoparticles

Many types of nanoparticles utilized in foods are fabricated using combinations of different ingredients, such as lipids, proteins and carbohydrates.^97,99,100^ For example, coacervates are typically formed by electrostatic complexation of oppositely charged proteins and polysaccharides. Lipid nanoparticles coated by nanolaminated layers can be formed by successive electrostatic deposition of oppositely charged biopolymers onto lipid droplet surfaces. These nanolaminated layers can have a pronounced influence on the GIT fate of the ingested particles. If the coatings remain intact, are impermeable, and indigestible in the GIT, then they may inhibit the digestion of the materials inside of the particles.

#### General comments

As mentioned earlier there have been few studies on the potential fate of organic nanoparticles after absorption, or of their potential toxicity. This is partly due to the challenges in analytically detecting organic nanoparticles within complex biological matrices that contain similar components (such as lipids, proteins, and polysaccharides). The development of suitable analytical techniques and protocols for this purpose would be a useful focus for future research.

## Characteristics of food nanoparticles

**Fig. 2:**
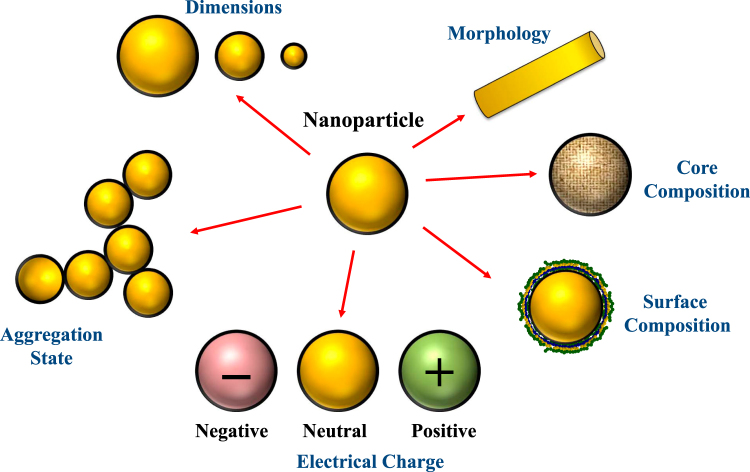
Food nanoparticles vary in particle characteristics, such as dimensions, morphology, composition, aggregation state, and charge

The nanoparticles found in food and beverage products vary considerably in their physiochemical and structural properties (Fig. 2), which determines their GIT fate and propensity to cause toxicity. Consequently, suitable analytical tools are required to characterize nanoparticle properties, which have been reviewed elsewhere.^7,101–103^ In this section, we focus on the ways that nanoparticles may vary.

### Composition

The nanoparticles found in foods may consist of inorganic (e.g., silver, titanium dioxide, silicon dioxide, iron oxide, and zinc oxide) and/or organic components (e.g., lipids, proteins, and carbohydrates). Nanoparticle composition plays a major role in determining their GIT fate. Lipids, proteins, and starches can be digested by proteases, lipases, and amylases in the mouth, stomach, small intestine, or colon. However, some organic substances used to fabricate food nanoparticles (such as dietary fibers and mineral oils) may not be digested in the upper GIT. Inorganic nanoparticles are also not digested in the GIT, but some of them may be fully or partially dissolved as a result of alterations in pH or dilution.^12,78^ Any nanoparticles that are not digested or absorbed in the upper GIT will reach the lower GIT where they may alter the microbiome.^12,34^ The ability of inorganic nanoparticles to produce toxicity is often associated with their chemical reactivity, which depends on their composition. For example, some inorganic nanoparticles dissolve and release ions that promote undesirable chemical or biochemical reactions (e.g., silver nanoparticles), whereas others are relatively inert (e.g., titanium dioxide nanoparticles).^104^

### Dimensions

Food nanoparticles vary considerably in their dimensions, ranging from a few nanometers (surfactant micelles) to a few hundred nanometers (lipid, protein, or carbohydrate nanoparticles), depending on the materials and processes used to create them.^8^ Nanoparticle dimensions influence their GIT fate and toxicity through a number of mechanisms.^6,105,106^ First, smaller nanoparticles are usually dissolved or digested more rapidly in GIT fluids than larger ones with similar compositions. Second, the ability of GIT components (such as digestive enzymes, phospholipids, bile salts, or mineral ions) to interact with nanoparticles is likely to increase as their size decreases because of the increase in surface area. Third, the penetration of nanoparticles through the mucus layer coating epithelium cells usually increases as their size decreases relative to the pore size of the biopolymer network. Fourth, the uptake of nanoparticles by intestinal epithelium cells through tight junctions, active transport, or passive transport mechanisms depends on particle size.

### Interfacial properties

The GIT fate of food-grade nanoparticles, and therefore their potential to have adverse health effects, is often influenced by their interfacial characteristics.^55,107,108^ Nanoparticles in foods and within the GIT are typically surrounded by a coating of adsorbed substances (sometimes referred to as a “corona”), which determines the electrical charge, hydrophobicity, thickness, digestibility, and chemical reactivity of the interface. These surface properties will determine the behavior of the nanoparticles in the GIT, such as their ability to penetrate biological barriers (such as the mucus layer or intestinal epithelium cells), their interaction with other components within the GIT (such as mucin, digestive enzymes, bile salts, mineral ions, or proteins), and their aggregation states (such as individual particles or clusters).

### Aggregation state

Food-grade nanoparticles may exist as isolated individual particles, or they may form clusters that vary in size, morphology, and strength. Typically, nanoparticles in clusters are held together by physical forces, such as Van der Waals, electrostatic, hydrogen bonding, and hydrophobic forces. The aggregation state of the nanoparticles is therefore often highly dependent on environmental conditions, such as pH, ionic strength, ingredient interactions, and mechanical forces. The dimensions of nanoparticle clusters may be much greater than the dimensions of the individual nanoparticles, which has a major impact on their GIT fate, such as their ability to move through the gastrointestinal fluids, mucus layer, or epithelium cells. Consequently, it is always important to determine the actual effective dimensions of the nanoparticles at the site of actions, rather than the dimensions of the original nanoparticles added in foods.

## Food matrix and GIT effects on nanoparticle characteristics and behaviors

A major factor that has been frequently ignored in the studies of the biological fate of ingested food nanoparticles is their interactions with various components within complex food matrices and GIT.^6,109^ Foodborne inorganic NPs are consumed as part of a food or beverage that may contain a variety of molecular and colloidal species that can interact with food nanoparticles and alter their biological fate. These interactions may occur within the food itself, or during the passage of the food nanoparticles through the GIT. The interaction of a food or GIT component with nanoparticles may alter their physicochemical properties in the GI tract and therefore their biological fate and function (e.g., absorption and toxicity). Indeed, the results of many previous studies have been highly limited because they used unrealistic test systems that ignored food matrix and GIT effects.

### Food matrix effects

Prior to ingestion, nanoparticles are typically dispersed within food matrices that vary considerably in their compositions, structures, and properties. Foods are composed of different types and levels of constituents (such as water, carbohydrates, fats, proteins, and minerals) that are assembled into different structural features (such as bulk phases, biological cells, polymers, droplets, bubbles, particles, and networks). Foods are produced using various processing operations (such as mixing/separation, cooling/heating, concentration/dilution, hydration/dehydration, and mechanical action). The physicochemical and structural properties of nanoparticles may therefore be changed considerably when they are dispersed in food products, which would play an important role in determining their subsequent GIT fate and toxicity. For example, the interfacial composition and properties of food-grade nanoparticles changes appreciably when they are added to foods or when they enter the GIT because of the adsorption of surface-active molecules from the surrounding environment.^110,111^ Moreover, it has been reported that certain flavonoids in foods can be tightly bound to the surface of inorganic nanoparticles.^112^ The interaction between these food components and nanoparticles may significantly alter the biological fate of these nanoparticles. Although knowledge of food matrix effects is critical for understanding the gastrointestinal fate of food nanoparticles, this important factor is currently ignored in most studies. Consequently, this should be an important focus for future research in this area.

### GIT effects

**Fig. 3:**
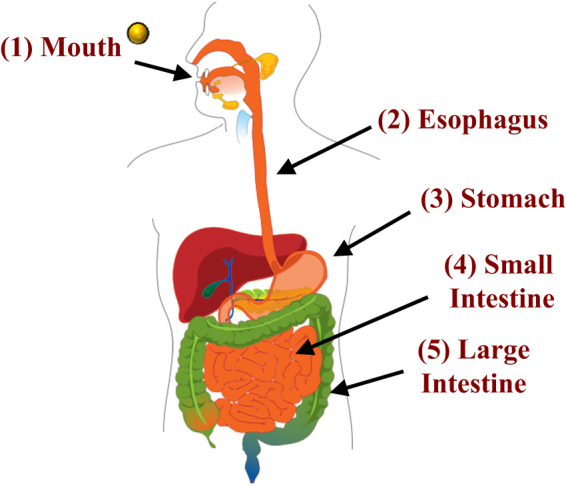
Schematic diagram of the different regions of the human gastrointestinal tract that nanoparticles must pass through. The diagram of the human body was taken from http://en.wikipedia.org/wiki/Digestive_tract (Copyright free). The characteristics of the different regions, such as pH and composition, and briefly described in the main text

After ingestion, nanoparticles travel through the complicated environment of the GIT before they are absorbed or exhibit their toxic effects^8^ (Fig. 3). Initially, nanoparticles pass through the mouth, which has an approximately neutral pH and encounters saliva that contains mucin, digestive enzymes (such as amylase), and electrolytes. The nanoparticles then move through the esophagus and into the stomach, where they are exposed to highly acidic gastric fluids (pH around 2–3) that contain digestive enzymes (gastric lipase and pepsin) and electrolytes. The nanoparticles then pass through the pylorus sphincter and enter the small intestine (pH around 5–7) where they are exposed to saliva fluids that contain bile salts, phospholipids, digestive enzymes (pancreatic lipase, proteases, and amylase), and electrolytes. If the nanoparticles are not absorbed in the upper GIT, then they will reach the colon (pH 6–7) where they will encounter colonic bacteria and undigested food components. If the nanoparticles are originally trapped within a food when they are ingested, then they may be released into the GIT fluids as the food matrix is disrupted and digested.^113–116^ The GIT region where they are released will therefore depend on the composition and structure of the food.

Some of the most important properties of GIT fluids that may alter nanoparticle characteristics are highlighted here:

pH and ionic strength: The pH and ionic composition of the gastrointestinal fluids depend on the nature of the food consumed and the specific GIT region (mouth, stomach, small intestine, or colon). These parameters determine the surface potential and electrostatic interactions of nanoparticles, which influences their aggregation state and interactions with other components.

Surface-active components: Gastrointestinal fluids contain surface-active components that may arise from the ingested food or from the GIT, such as surfactants, proteins, bile salts, phospholipids, and FFAs. These surface-active components may adsorb to nanoparticle surfaces and alter their interfacial properties and subsequently their biological fate. For example, the interfacial properties of inorganic nanoparticles is changed appreciably when they enter the GIT, which impacts cellular and tissue response to the nanoparticles.^117,118^ Even after cellular uptake, inorganic nanoparticles may interact with cellular and/or blood proteins, which can further modify their biological fate.^76,119–121^

Enzyme activity: Gastrointestinal fluids contain digestive and metabolic enzymes that may change the properties of certain types of nanoparticles. For example, nanoparticles containing starches, proteins, or lipid may be digested by amylases, proteases, or lipases. Consequently, the properties of the nanoparticles reaching specific regions within the GIT may be very different from those of the ingested nanoparticles.

Biopolymers: Gastrointestinal fluids contain biopolymers that may also alter the properties of nanoparticles. These biopolymers may arise from the ingested food or be secreted by the GIT, e.g., proteins, polysaccharides, and glycoproteins. Biopolymers may adsorb to nanoparticle surfaces and change their interfacial properties, or they may alter their aggregation state by promoting or opposing flocculation. For example, adsorbing biopolymers may promote bridging flocculation, whereas non-adsorbing biopolymers may promote depletion flocculation.

Mucus layer and GIT surfaces: The GIT consists of a series of tubes and chambers with complex surface morphologies, e.g., villi and micro-villi. These surfaces are usually coated by a thin layer of mucus that acts as a barrier to the direct interaction of nanoparticles with the underlying epithelial cells.^105^ The nature of the mucus layer and GIT surfaces depend on their location within the human body (e.g., mouth, stomach, small intestine, and colon). Ingested nanoparticles may adhere to, travel through, or be adsorbed by the mucus layer and GIT surfaces depending on their characteristics.

GIT microbiota: Numerous species of bacteria reside within the human GIT, with the majority populating the large intestine. GIT bacteria may generate products that change the properties of ingested nanoparticles (such as enzymes or biopolymers). Conversely, ingested nanoparticles may change the properties of GIT bacteria.^12^ In particular, many types of inorganic nanoparticles have antimicrobial properties and may therefore alter the balance of different bacterial species in the colon, potentially leading to adverse health effects.^12,13^ The presence of inorganic nanoparticles in the colon may also impact the bioactivity of other substances (such as antibiotics), possibly by damaging bacterial cell walls.^122^

Mechanical forces: Ingested nanoparticles are containing within gastrointestinal fluids that are subjected to various kinds of mechanical forces as they pass through the GIT (such as mastication in the mouth, peristalsis in the esophagus and small intestine, and churning in the stomach), which may alter the properties of the nanoparticles. In particular, mechanical forces may alter the aggregation state of nanoparticles by breaking down weakly flocculated systems.

As a result of these factors, the properties of nanoparticles are changed appreciably as they pass through the GIT, which will alter their GIT fate and potential toxicity. For example, there may be changes in the composition, dimensions, surface properties, physical state, and aggregation state of nanoparticles (Fig. 2), which should be taken into account when establishing their potential toxicity. The importance of this effect is highlighted by a recent study, which reported that the interfacial properties of inorganic (magnetite) nanoparticles co-ingested with bread were altered in a way that promoted their uptake by intestinal epithelium cells.^123^ Another in vitro study showed that the presence of a digested food matrix enhanced the absorption of silver nanoparticles by intestinal epithelium cells.^124^ These findings demonstrated that the characteristics of the nanoparticles inside the GIT may be appreciably different to those of the original (pristine) nanoparticles, which is often ignored in biological fate and toxicity assessments of food nanoparticles potentially leading to unrealistic and misleading results.

## Potential mechanisms of action of nanoparticle toxicity

Ingested nanoparticles may cause toxicity due to numerous physicochemical and physiological mechanisms depending on their compositions, structures, and properties. This section highlights some of the most important mechanisms of nanoparticle toxicity.

### Interference with normal GIT function

The presence of nanoparticles in the gastrointestinal fluids could interfere with normal GIT functions. The small size of nanoparticles means they have a high specific surface area, which offers a large area for adsorption of any surface-active components in the GIT. For example, digestive or metabolic enzymes could adsorb to nanoparticle surfaces thereby altering their normal GIT function. Many globular proteins are denatured after adsorption to particle surfaces due to the change in their thermodynamic environment, which could lead to a reduction in the catalytic activity of some enzymes. Consequently, high levels of nanoparticles could reduce the rate or extent of starch, lipid, or protein digestion within the GIT. This effect is mainly important for inorganic nanoparticles, but it may also be important for some organic nanoparticles (particularly indigestible ones).

An estimate of the potential magnitude of above effect can be obtained by considering the impact of inorganic nanoparticles on the digestion of lipid droplets. In this mixed system, the lipase molecules could adsorb to the surfaces of either the lipid droplets or the nanoparticles. The fraction of the total surface area (nanoparticles + droplets) due to the droplets is given by the following equation:

$${\mathrm{\Omega }} = \frac{{\phi _1}}{{\phi _1 + \phi _2d_1/d_2}}$$

Here *ϕ*_1_ and *ϕ*_2_ are the volume fractions and *d*_1_ and *d*_2_ are the diameters of the lipid droplets and inorganic nanoparticles, respectively. This equation shows that the fraction of lipid droplet surface area decreases as the concentration of inorganic nanoparticles increases or their diameter decreases. As an example, assume there is 5% lipid droplets in the small intestine (*ϕ*_1_ = 0.05) as well as some inorganic nanoparticles (*d*_2_ = 100 nm). The concentration of inorganic nanoparticles required to reduce the amount of lipase adsorbed to the lipid droplet surfaces by 50% would be:

$$\phi _2 = \frac{{d_2}}{{d_1}}\phi _1$$

Thus, there would have to be inorganic nanoparticle levels of 5, 2.5, 1, 0.5, 0.25, 0.1 and 0.05% for lipid droplets with diameters of 100, 200, 500, 1000, 2000, 5000, and 10,000 nm, respectively. Typically, the concentration of inorganic nanoparticles in the small intestine is likely to be a fraction of a percent, and so this effect is only likely to be important for relatively large lipid droplets at relatively low concentrations. In addition, the effect is likely to be less than predicted by the above equations for a number of reasons: (i) the inorganic nanoparticles may aggregate in the GIT, which reduces their exposed surface area; (ii) the lipase molecules may adsorb more strongly to the lipid droplet surfaces than to the inorganic nanoparticles surfaces; (iii) there may be other surface-active substances in the GIT that compete with the lipase for the surfaces of the inorganic nanoparticles. Indeed, recent experiments in our laboratory have shown that mixing titanium dioxide nanoparticles with lipid droplets only caused a slight decrease in lipid digestion. Other substances in the GIT fluids involved in the digestion of macronutrients may also adsorb to the surfaces of inorganic nanoparticles, such as bile salts and phospholipids.

There has been little research in this area, and so it is difficult to assess any potential harmful effects associated with this mechanism. At the worst, one might expect that there would be a reduction in the rate of lipid, protein, or starch digestion, but that these components would eventually be fully digested due to the bodies’ ability to secrete additional enzymes and other digestive components when needed. Due to the relatively low levels of inorganic nanoparticles normally ingested, the authors do not anticipate that this mechanism will be a major health concern.

Some types of inorganic nanoparticles may also be able to physically disrupt important structures within the GIT, such as the tight junctions or microvilli, thereby altering normal nutrient absorption and the protective function of the epithelium cells.^12^ The presence of nanoparticles in the GIT may also stimulate an immune response, which could have adverse effects on human health, and so this possibility should be tested for food-grade nanoparticles.^125^

### Accumulation within specific tissues

The results of animal studies suggest that certain types of ingested nanoparticles are absorbed within the GIT and accumulate in numerous tissues.^15^ Presumably, these nanoparticles travel across the mucus layer and are then absorbed by active or passive transport mechanisms. After they have been absorbed into the cells, the nanoparticles may be metabolized, transferred out of the cells, or accumulate within the cells. These processes depend on nanoparticle characteristics such as composition, dimensions, morphology, aggregation state and interfacial properties. The accumulation of nanoparticles within specific tissues may lead to long-term problems if they exhibit toxic effects above a certain accumulation threshold. This mechanism of action is likely to be most important for inorganic nanoparticles that are bio-persistent (not normally digested or metabolized in GIT).

### Cytotoxicity and cellular malfunction

Nanoparticles may produce toxicity in cells through a variety of different mechanisms, depending on their composition and structure.^12^ One of the most important factors contributing to the toxicity of inorganic nanoparticles is their ability to generate ROS, such as singlet oxygen, superoxide, hydrogen peroxide and hydroxyl radicals.^49^ These ROS may then cause damage to cell membranes, organelles, and the nucleus by interacting with lipids, proteins, or nucleic acids.^15,33,49^ As a result, many biochemical functions required to maintain cell viability, such as ATP production, DNA replication, and gene expression, may be adversely affected.^33^ A number of studies have reported the ability of inorganic nanoparticles to increase the generation of ROS in cells and to produce cytotoxicity, including silicon dioxide nanoparticles,^81^ ZnO nanoparticles,^36,126,127^ and silver nanoparticles.^15^ Some inorganic nanoparticles produce toxicity by generating ions (such as Ag^+^ from silver nanoparticles or Zn ^2+^ from zinc oxide nanoparticles) that interact with the normal functioning cellular components (such as proteins, nucleic acids, or lipids) required to maintain biochemical processes. These mechanisms of action are most likely to be important for inorganic nanoparticles that are absorbed by the intestinal cells, since most organic nanoparticles are digested before being absorbed. However, it is still unclear about the extent to which inorganic nanoparticles would produce cytotoxicity when they are consumed as part of a complex diet under normal conditions.

### Altered location of bioactive release

The encapsulation of bioactive agents (nutrients or nutraceuticals) within nanoparticles may alter the location of their release and absorption within the GIT. For example, a bioactive agent that is normally released in the mouth, stomach, or small intestine could be released within the colon. As a result, the physiological response and biological impact of the bioactive agent may be altered by nanoencapsulation, which could have potentially adverse health effects. For example, the encapsulation of digestible lipids within nanolaminated dietary fiber coatings may inhibit the rate and extent of lipid digestion in the upper GIT,^92^ so that high levels of undigested lipids reach the colon. These lipids may then be fermented by the colonic bacteria, which could cause gastrointestinal problems. Alternatively, an antimicrobial agent may be encapsulated within a nanoparticle that is not digested within the upper GIT, so that it reaches the colon, where it could alter the nature of the colonic microflora, which could again have adverse health effects.

To the authors knowledge there have been few specific studies on this mechanism of potential toxicity of nanomaterials, and further work in clearly needed. These effects are likely to be highly system-specific, depending on the nature of the encapsulated bioactive and nanoparticle used, and would therefore need to be established on a case-by-case basis.

### Enhancement of oral bioavailability

**Fig. 4:**
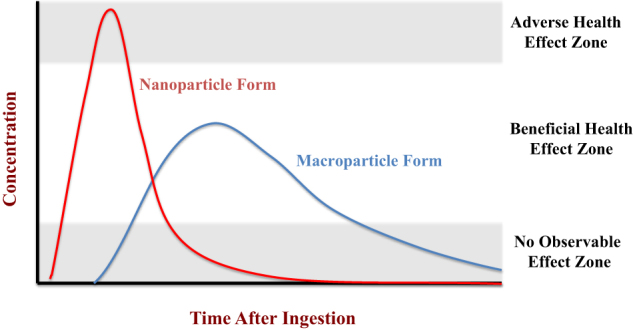
Schematic representation of the change in the concentration of a bioactive substance in the systemic circulation after ingestion. Changing the dimensions of the particles used to deliver a bioactive substance may alter its release, transport, absorption, and metabolism in the GIT, thereby altering its concentration–time profile in the systemic circulation

One of the most widely studied applications of nanotechnology in the food industry is for the encapsulation and delivery of hydrophobic bioactive agents, such as certain nutrients and nutraceuticals.^97^ Numerous in vitro and in vivo studies have shown that delivering these bioactive agents within nanoparticles (rather than within larger particles or in bulk phases) can greatly increase their bioavailability. For example, nanoemulsions have been shown to increase the bioaccessibility or bioavailability of carotenoids,^128^ curcumin,^129^ coenzyme Q10,^130,131^ ω-3 fatty acids,^132^ and fat-soluble vitamins.^133^ There are a number of different physicochemical mechanisms that may be responsible for this improvement. In particular, the nanoparticles may increase the bioaccessibility, chemical stability, and/or absorption of the encapsulated bioactive agents.^87^ In general, nanoparticles tend to be digested or dissolved more rapidly in the GIT and/or release any encapsulated components more rapidly because of their small size and high surface area. This will lead to differences in the pharmacokinetics of the bioactive agents within the systemic circulation (Fig. 4). A change in the exposure level of bioactive agents within the blood could have potentially adverse health effects. The biological effects of many bioactive agents depend on their exposure levels in the blood and specific tissues. If the exposure level is too low, then the bioactive agent will have little biological impact. If the exposure level is too high, then it may be toxic. Thus the concentration should be within a certain intermediate level to have the most beneficial biological effects. This effect is likely to be highly system-dependent. In particular, it will depend on the toxicity profile of the bioactive agent. Some bioactive agents can be consumed at relatively high levels and have little toxicity, and therefore the ability of nanoparticles to boost their bioavailability should not have any adverse consequences. On the other hand, boosting the bioavailability of some bioactive agents could cause health problems. Vitamin E (a mixture of tocopherols and tocotrienols) is essential for maintaining human health and performance. However, consumption of high doses of vitamin E may increase the risk of various chronic diseases.^134^ Much of the studies establishing the upper limits for the adverse health effects of bioactive agents have not taken into account the nature of the delivery systems used. Thus, the level where toxic effects are observed could be appreciably lower in cases where nanoparticle delivery systems greatly increase the bioavailability of the bioactive agents being tested.

Nanoparticles may increase the bioavailability of bioactive agents through two different approaches: delivery systems or excipient systems.^135^ In delivery systems, the bioactive agent is encapsulated within the nanoparticles. In excipient systems, a food containing nanoparticles (excipient food) is consumed with a bioactive-rich food (such as fruits or vegetables). In both cases, the delivery or excipient system is specifically designed to increase the bioavailability of the bioactive agents by increasing the bioaccessibility or absorption, or by modulating any transformations (such as chemical or biochemical reactions) of the bioactive agents in the GIT.

### Enhancement of pesticide bioavailability

The ability of nanoparticles to greatly increase the oral bioavailability of hydrophobic substances could also have adverse health effects by promoting the uptake of undesirable non-polar substances in foods, such as certain pesticides and hormones. For example, a food product that contains lipid nanoparticles (such as a beverage, sauce, dressing, or cream) may increase the bioavailability of hydrophobic pesticides on fruits or vegetables consumed with them. At present there is little information on this mechanism, and there is a need for further studies. This mechanism is likely to be most important for foods containing lipid nanoparticles (nanoemulsions) that are consumed with foods potentially containing high levels of hydrophobic pesticides or hormones (such as some fruits and vegetables).

### Interference with gut microbiota

Nanoparticles that reach the colon may interact with colonic bacteria and alter their viability, thereby changing the relative proportions of different bacterial species present.^12,13^ The type of bacteria populating the human colon is known to play a major role in human health and wellbeing.^136,137^ Consequently, any change in the gut microbiota due to the presence of food-grade nanoparticles could have adverse health effects. This is an important area that requires further research to determine the impact of specific nanoparticle characteristics on the gut microbiota and the resulting health implications.

## Current status and future recommendations

The study of the potential toxic effects of food-grade nanoparticles has increased considerably in the past few years. Researchers from many disciplines have studied the potentially toxic effects of various kinds of organic and inorganic food nanoparticles. The results of these studies have led to considerable insights into the type of food nanoparticles that may cause adverse health effects, as well as into the possible physicochemical and physiological mechanisms involved. Nevertheless, there is still a considerable amount of confusion in this area and many contradictory results, with some studies suggesting that a particular type of nanoparticle does not produce toxicity, whereas other studies showing that they are toxic. There are a number of reasons for these discrepancies:

- Nanoparticles with different physicochemical properties were used, such as internal composition, surface composition, physical state, crystal form, dimensions, shape, aggregation state, and dose. In many studies, the properties of the nanoparticles were not adequately characterized or reported.
- Different test methods were used to establish their potential toxicity, such as physicochemical, cell culture, microbial, animal, and human feeding studies. Moreover, the test methods used often vary considerably from laboratory-to-laboratory, which makes it difficult to directly compare results.
- The effects of the dietary patterns, food matrix, and passage through the GIT were often ignored. These factors may have a major impact on nanoparticle characteristics, behavior, and toxicity.

It is clear that standardized methods need to be developed to adequately test nanoparticle toxicity under reproducible and realistic conditions.

## Conclusions

There is considerable interest in utilizing both organic and inorganic nanoparticles within foods because of their potential for improving food quality, safety, or nutritional attributes. However, the small size of nanoparticles means that they may behave differently within the human body than the larger particles or bulk materials conventionally utilized as food ingredients. As a result, there is a need to better understand the GIT fate of ingested nanoparticles and to characterize their potential toxicity. At present there is a relatively poor understanding of the GIT fate and toxicity of most types of food-grade nanoparticles, and it is not possible to make a single general recommendation about the safety of all nanoparticle types. Instead, the safety of nanoparticles should be judged on a case-by-case basis depending on the nature of the nanoparticles, as well as the properties of the food matrix they are dispersed within.

In the authors’ experience, different mechanisms of action are likely to be more or less important for different types of nanoparticles. For inorganic nanoparticles, their ability to be absorbed by the body, accumulate in certain tissues, and produce cytotoxicity are likely to be the most important mechanisms. For organic nanoparticles, their ability to enhance the bioavailability of potentially toxic substances (such as pesticides or hormones) or substances that are only toxic at high levels (such as certain fat-soluble vitamins) are likely to be more important mechanisms. Nevertheless, more research is needed to establish the potential magnitude and importance of these effects.

## References

[1] Chaudhry Q (2008). Applications and implications of nanotechnologies for the food sector. Food Addit. Contam. Part A Chem. Anal. Control. Expo. Risk Assess..

[2] Sozer N, Kokini JL (2009). Nanotechnology and its applications in the food sector. Trends Biotechnol..

[3] Livney YD (2010). Milk proteins as vehicles for bioactives. Curr. Opin. Colloid Interface Sci..

[4] Holt C, de Kruif CG, Tuinier R, Timmins PA (2003). Substructure of bovine casein micelles by small-angle X-ray and neutron scattering. Colloid Surf. A.

[5] Iwanaga D (2007). Extraction and characterization of oil bodies from soy beans: A natural source of pre-emulsified soybean oil. J. Agric. Food Chem..

[6] Bellmann S (2015). Mammalian gastrointestinal tract parameters modulating the integrity, surface properties, and absorption of food-relevant nanomaterials. Nanomed. Nanobiotech..

[7] Szakal C (2014). Measurement of nanomaterials in foods: integrative consideration of challenges and future prospects. ACS Nano.

[8] Yada RY (2014). Engineered nanoscale food ingredients: evaluation of current knowledge on material characteristics relevant to uptake from the gastrointestinal tract. Comp. Rev. Food Sci. Food Saf..

[9] Gupta A, Eral HB, Hatton TA, Doyle PS (2016). Nanoemulsions: formation, properties and applications. Soft Matter.

[10] Fellows, P. J. *Food Processing Technology* 4th edn (Woodhead Publishing, 2017).

[11] Buzea C (2007). Pacheco, II & Robbie, K. Nanomaterials and nanoparticles: sources and toxicity. Biointerphases.

[12] Frohlich, E. E. & Frohlich, E. Cytotoxicity of nanoparticles contained in food on intestinal cells and the gut microbiota. *Int. J. Mol. Sci*. **17**, 1–22 (2016). 10.3390/ijms17040509 PMC4848965 27058534

[13] Pietroiusti A, Magrini A, Campagnolo L (2016). New frontiers in nanotoxicology: Gut microbiota/microbiome-mediated effects of engineered nanomaterials. Toxicol. Appl. Pharma..

[14] Hajipour MJ (2012). Antibacterial properties of nanoparticles. Trends Biotechnol..

[15] Gaillet S, Rouanet JM (2015). Silver nanoparticles: their potential toxic effects after oral exposure and underlying mechanisms–a review. Food Chem. Toxicol..

[16] Pulit-Prociak J, Stoklosa K, Banach M (2015). Nanosilver products and toxicity. Environ. Chem. Lett..

[17] Echegoyen Y, Nerin C (2013). Nanoparticle release from nano-silver antimicrobial food containers. Food Chem. Toxicol..

[18] Pugliara. A (2016). Assessing bio-available silver released from silver nanoparticles embedded in silica layers using the green algae Chlamydomonas reinhardtii as bio-sensors. Sci. Total Environ..

[19] Mackevica, A., Olsson, M. E. & Hansen, S. F. Silver nanoparticle release from commercially available plastic food containers into food simulants. *J. Nanopart. Res*. **18**, 1–11 (2016).

[20] Loza K, Sengstock C, Chernousova S, Koller M, Epple M (2014). The predominant species of ionic silver in biological media is colloidally dispersed nanoparticulate silver chloride. RSC Adv..

[21] Cha K (2008). Comparison of acute responses of mice livers to short-term exposure to nano-sized or micro-sized silver particles. Biotech. Lett..

[22] Kim YS (2010). Subchronic oral toxicity of silver nanoparticles. *Part*. Fibre Toxicol..

[23] Shahare B, Yashpal M (2013). Toxic effects of repeated oral exposure of silver nanoparticles on small intestine mucosa of mice. Toxicol. Mech. Methods.

[24] Jeong GN (2010). Histochemical study of intestinal mucins after administration of silver nanoparticles in Sprague-Dawley rats. Arch. Toxicol..

[25] Hendrickson OD (2016). Toxicity of nanosilver in intragastric studies: Biodistribution and metabolic effects. Toxicol. Lett..

[26] Kim YS (2008). Twenty-eight-day oral toxicity, genotoxicity, and gender-related tissue distribution of silver nanoparticles in Sprague-Dawley rats. Inhal. Toxicol..

[27] Park EJ (2010). Repeated-dose toxicity and inflammatory responses in mice by oral administration of silver nanoparticles. Environ. Toxicol. Pharmacol..

[28] Garcia T (2010). Oral subchronic exposure to silver nanoparticles in rats. Food Chem. Toxicol..

[29] Loeschner K (2011). Distribution of silver in rats following 28 days of repeated oral exposure to silver nanoparticles or silver acetate. Part. Fiber Toxicol..

[30] Chen N (2016). Toxicological effects of Caco-2 cells following short-term and long-term exposure to Ag nanoparticles. Int. J. Mol. Sci..

[31] Georgantzopoulou A (2016). Effects of silver nanoparticles and ions on a co-culture model for the gastrointestinal epithelium. Part. Fiber Toxicol..

[32] Kim TH (2012). Size-dependent cellular toxicity of silver nanoparticles. J. Biomed. Mat. Res. A.

[33] Sharma VK, Siskova KM, Zboril R, Gardea-Torresdey JL (2014). Organic-coated silver nanoparticles in biological and environmental conditions: fate, stability and toxicity. Adv. Coll. Int. Sci..

[34] Williams K (2015). Effects of subchronic exposure of silver nanoparticles on intestinal microbiota and gut-associated immune responses in the ileum of Sprague-Dawley rats. Nanotoxicology.

[35] Lichtenstein D (2015). Impact of food components during in vitro digestion of silver nanoparticles on cellular uptake and cytotoxicity in intestinal cells. Biol. Chem..

[36] Wang YL (2014). A combined toxicity study of zinc oxide nanoparticles and vitamin C in food additives. Nanoscale.

[37] Sirelkhatim A (2015). Review on zinc oxide nanoparticles: antibacterial activity and toxicity mechanism. Nano Micro Lett..

[38] EFSA. (2016). Safety assessment of the substance zinc oxide, nanoparticles, for use in food contact materials. EFSDA J..

[39] Bumbudsanpharoke N, Ko S (2015). Nano-food packaging: an overview of market, migration research, and safety regulations. J. Food Sci..

[40] Vandebriel RJ, De Jong WH (2012). A review of mammalian toxicity of ZnO nanoparticles. Nanotech. Sci. Appl..

[41] Pasupuleti S (2012). Toxicity of zinc oxide nanoparticles through oral route. Toxicol. Ind. Health.

[42] Wang B (2008). Acute toxicological impact of nano-and submicro-scaled zinc oxide powder on healthy adult mice. J. Nanopart. Res..

[43] Wang H, Du LJ, Song ZM, Chen XX (2013). Progress in the characterization and safety evaluation of engineered inorganic nanomaterials in food. Nanomedicine.

[44] Esmaeillou M, Moharamnejad M, Hsankhani R, Tehrani AA, Maadi H (2013). Toxicity of ZnO nanoparticles in healthy adult mice. Env. Toxicol. Pharmacol..

[45] Kang TS (2015). Cytotoxicity of zinc oxide nanoparticles and silver nanoparticles in human epithelial colorectal adenocarcinoma cells. Food Sci. Technol..

[46] Bacchetta R (2014). Evidence and uptake routes for zinc oxide nanoparticles through the gastrointestinal barrier in Xenopus laevis. Nanotoxicology.

[47] Raspopov RV, Trushina EN, Gmoshinsky IV, Khotimchenko SA (2011). Bioavailability of nanoparticles of ferric oxide when used in nutrition. Experimental results in rats. Vopr. Pitan..

[48] Zimmermann MB, Hilty FM (2011). Nanocompounds of iron and zinc: their potential in nutrition. Nanoscale.

[49] Wu HH, Yin JJ, Wamer WG, Zeng MY, Lo YM (2014). Reactive oxygen species-related activities of nano-iron metal and nano-iron oxides. J. Food Drug. Anal..

[50] Hilty FM (2010). Iron from nanocompounds containing iron and zinc is highly bioavailable in rats without tissue accumulation. Nat. Nanotech..

[51] WHO in *Safety Evaluation of Certain Food Additives and Contaminants* Vol. 44, 1–2 (World Health Organization, 2000). 11286008

[52] Fulgoni VL, Keast DR, Bailey RL, Dwyer J (2011). Foods, fortificants, and supplements: where do Americans get their nutrients?. J. Nutr..

[53] Patil US (2015). in vitro/in vivo toxicity evaluation and quantification of iron oxide nanoparticles. Int. J. Mol. Sci..

[54] Yun JW (2015). Comparative toxicity of silicon dioxide, silver and iron oxide nanoparticles after repeated oral administration to rats. J. Appl. Toxicol..

[55] Weir A, Westerhoff P, Fabricius L, Hristovski K, von Goetz N (2012). Titanium dioxide nanoparticles in food and personal care products. Environ. Sci. Technol..

[56] Jovanović B (2015). Critical review of public health regulations of titanium dioxide, a human food additive. Integr. Environ. Assess. Manag..

[57] Warheit DB, Brown SC, Donner EM (2015). Acute and subchronic oral toxicity studies in rats with nanoscale and pigment grade titanium dioxide particles. Food Chem. Toxicol..

[58] Weir A, Westerhoff P, Fabricius L, Hristovski K, von Goetz N (2012). Titanium dioxide nanoparticles in food and personal care products. Environ. Sci. Technol..

[59] Chen XX (2013). Characterization and preliminary toxicity assay of nano-titanium dioxide additive in sugar-coated chewing gum. Small.

[60] Yang Y (2014). Characterization of food-grade titanium dioxide: the presence of nanosized particles. Environ. Sci. Technol..

[61] Wang J (2007). Acute toxicity and biodistribution of different sized titanium dioxide particles in mice after oral administration. Toxicol. Lett..

[62] Duan Y (2010). Toxicological characteristics of nanoparticulate anatase titanium dioxide in mice. Biomaterials.

[63] Nogueira CM (2012). Titanium dioxide induced inflammation in the small intestine. World J. Gastroenterol..

[64] Bu Q (2010). NMR-based metabonomic study of the sub-acute toxicity of titanium dioxide nanoparticles in rats after oral administration. Nanotechnology.

[65] Cho WS (2013). Comparative absorption, distribution, and excretion of titanium dioxide and zinc oxide nanoparticles after repeated oral administration. Part. Fibre Toxicol..

[66] Wang Y (2013). Susceptibility of young and adult rats to the oral toxicity of titanium dioxide nanoparticles. Small.

[67] Tada-Oikawa S (2016). Titanium dioxide particle type and concentration influence the inflammatory response in Caco-2 cells. Int. J. Mol. Sci..

[68] Song ZM (2015). Biological effect of food additive titanium dioxide nanoparticles on intestine: an in vitro study. J. Appl. Toxicol..

[69] Brun E (2014). Titanium dioxide nanoparticle impact and translocation through ex vivo, in vivo and in vitro gut epithelia. Part. Fiber Toxicol..

[70] Chalew TEA, Schwab KJ (2013). Toxicity of commercially available engineered nanoparticles to Caco-2 and SW480 human intestinal epithelial cells. Cell Biol. Toxicol..

[71] Gerloff K, Albrecht C, Boots AW, Forster I, Schins RPF (2009). Cytotoxicity and oxidative DNA damage by nanoparticles in human intestinal Caco-2 cells. Nanotoxicology.

[72] Dorier M (2015). Impact of anatase and rutile titanium dioxide nanoparticles on uptake carriers and efflux pumps in Caco-2 gut epithelial cells. Nanoscale.

[73] Kruger K, Cossais F, Neve H, Klempt M (2014). Titanium dioxide nanoparticles activate IL8-related inflammatory pathways in human colonic epithelial Caco-2 cells. J. Nanopart. Res..

[74] Dorier M (2015). Impact of anatase and rutile titanium dioxide nanoparticles on uptake carriers and efflux pumps in Caco-2 gut epithelial cells. Nanoscale.

[75] Gerloff K (2012). Distinctive toxicity of TiO2 rutile/anatase mixed phase nanoparticles on Caco-2 cells. Chem. Res. Toxicol..

[76] Monopoli MP (2011). Physical−chemical aspects of protein corona: relevance to in vitro and in vivo biological impacts of nanoparticles. J. Am. Chem. Soc..

[77] Lesniak A (2013). Nanoparticle adhesion to the cell membrane and its effect on nanoparticle uptake efficiency. J. Am. Chem. Soc..

[78] Dekkers S (2011). Presence and risks of nanosilica in food products. Nanotoxicology.

[79] Peters R (2012). Presence of nano-sized silica during in vitro digestion of foods containing silica as a food additive. ACS Nano.

[80] Yang Y (2016). Survey of food-grade silica dioxide nanomaterial occurrence, characterization, human gut impacts and fate across its lifecycle. Sci. Total Environ..

[81] Athinarayanan J, Periasamy VS, Alsaif MA, Al-Warthan AA, Alshatwi AA (2014). Presence of nanosilica (E551) in commercial food products: TNF-mediated oxidative stress and altered cell cycle progression in human lung fibroblast cells. Cell. Biol. Toxicol..

[82] van Kesteren PCE (2015). Novel insights into the risk assessment of the nanomaterial synthetic amorphous silica, additive E551, in food. Nanotoxicology.

[83] So SJ, Jang IS, Han CS (2008). Effect of micro/nano silica particle feeding for mice. J. Nanosci. Nanotech..

[84] McClements DJ (2013). Edible lipid nanoparticles: digestion, absorption, and potential toxicity. Prog. Lipid Res..

[85] Piorkowski DT, McClements DJ (2014). Beverage emulsions: recent developments in formulation, production, and applications. Food Hydrocoll..

[86] Livney YD (2015). Nanostructured delivery systems in food: latest developments and potential future directions. Curr. Opin. Food Sci..

[87] McClements DJ (2015). Reduced-fat foods: the complex science of developing diet-based strategies for tackling overweight and obesity. Adv. Nutr..

[88] Shin GH, Kim JT, Park HJ (2015). Recent developments in nanoformulations of lipophilic functional foods. Trends Food Sci. Tech..

[89] Yao M, McClements DJ, Xiao H (2015). Improving oral bioavailability of nutraceuticals by engineered nanoparticle-based delivery systems. Curr. Opin. Food Sci..

[90] McClements DJ, Rao J (2011). Food-grade nanoemulsions: formulation, fabrication, properties, performance, biological fate, and potential toxicity. Crit. Rev. Food Sci. Nutr..

[91] McClements DJ, Xiao H (2012). Potential biological fate of ingested nanoemulsions: influence of particle characteristics. Food Funct..

[92] McClements DJ (2010). Design of nano-laminated coatings to control bioavailability of lipophilic food components. J. Food Sci..

[93] Oftedal OT (2012). The evolution of milk secretion and its ancient origins. Animal.

[94] Rajendran S, Udenigwe CC, Yada RY (2016). Nanochemistry of protein-based delivery agents. Front. Chem..

[95] Davidov-Pardo G, Joye IJ, McClements DJ (2015). Food-grade protein-based nanoparticles and microparticles for bioactive delivery: fabrication, characterization, and utilization. Adv. Protein Chem. Struct. Biol..

[96] Myrick JM, Vendra VK, Krishnan S (2014). Self-assembled polysaccharide nanostructures for controlled-release applications. Nanotech. Rev..

[97] Joye IJ, Davidov-Pardo G, McClements DJ (2014). Nanotechnology for increased micronutrient bioavailability. Trends Food Sci. Tech..

[98] Le Corre D, Bras J, Dufresne A (2010). Starch nanoparticles: a review. Biomacromolecules.

[99] Jones OG, McClements DJ (2010). Functional biopolymer particles: design, fabrication, and applications. Comp. Rev. Food Sci. Food Saf..

[100] McClements DJ (2012). Advances in fabrication of emulsions with enhanced functionality using structural design principles. Curr. Opin. Colloid Int. Sci..

[101] McClements J, McClements DJ (2016). Standardization of nanoparticle characterization: methods for testing properties, stability, and functionality of edible nanoparticles. Crit. Rev. Food Sci. Nutr..

[102] Wang H, Du LJ, Song ZM, Chen XX (2013). Progress in the characterization and safety evaluation of engineered inorganic nanomaterials in food. Nanomedicine.

[103] Singh G, Stephan C, Westerhoff P, Carlander D, Duncan TV (2014). Measurement methods to detect, characterize, and quantify engineered nanomaterials in foods. Comp. Rev. Food Sci. Food Saf..

[104] Pradeep T, Anshup (2009). Noble metal nanoparticles for water purification: a critical review. Thin Solid Films..

[105] Ensign LM, Cone R, Hanes J (2012). Oral drug delivery with polymeric nanoparticles: the gastrointestinal mucus barriers. Adv. Drug Deliv. Rev..

[106] Frohlich E, Roblegg E (2012). Models for oral uptake of nanoparticles in consumer products. Toxicology.

[107] Magnuson BA, Jonaitis TS, Card JW (2011). A brief review of the occurrence, use, and safety of food-related nanomaterials. J. Food Sci..

[108] Powell JJ, Faria N, Thomas-McKay E, Pele LC (2010). Origin and fate of dietary nanoparticles and microparticles in the gastrointestinal tract. J. Autoimmun..

[109] McClements DJ (2016). The role of the food matrix and gastrointestinal tract in the assessment ofbiological properties of ingested engineered nanomaterials (iENMs): state of the science and knowledge gaps. NanoImpact.

[110] Saptarshi SR, Duschl A, Lopata AL (2013). Interaction of nanoparticles with proteins: relation to bio-reactivity of the nanoparticle. J. Nanobiotech..

[111] Monopoli MP, Åberg C, Salvati A, Dawson KA (2012). Biomolecular coronas provide the biological identity of nanosized materials. Nat. Nanotechn..

[112] Cao X (2016). Characterization of the interactions between titanium dioxide nanoparticles and polymethoxyflavones using surface-enhanced Raman spectroscopy. J. Agric. Food Chem..

[113] Blijdenstein TBJ, van der Linden E, van Vliet T, van Aken GA (2004). Scaling behavior of delayed demixing, rheology, and microstructure of emulsions flocculated by depletion and bridging. Langmuir.

[114] de Hoog EHA, Prinz JF, Huntjens L, Dresselhuis DM, van Aken GA (2006). Lubrication of oral surfaces by food emulsions: the importance of surface characteristics. J. Food Sci..

[115] Silletti E, Vingerhoeds MH, Norde W, Van Aken GA (2007). The role of electrostatics in saliva-induced emulsion flocculation. Food Hydrocoll..

[116] Vingerhoeds MH, Blijdenstein TBJ, Zoet FD, van Aken GA (2005). Emulsion flocculation induced by saliva and mucin. Food Hydrocoll..

[117] Mahmoudi M (2011). Protein−nanoparticle interactions: opportunities and challenges. Chem. Rev..

[118] Giri K (2014). Understanding protein–nanoparticle interaction: a new gateway to disease therapeutics. Bioconjug. Chem..

[119] Lacerda SHDP (2009). Interaction of gold nanoparticles with common human blood proteins. ACS Nano.

[120] Dobrovolskaia MA (2009). Interaction of colloidal gold nanoparticles with human blood: effects on particle size and analysis of plasma protein binding profiles. Nanomed. Nanotechnol. Biol. Med..

[121] Aggarwal P, Hall JB, McLeland CB, Dobrovolskaia MA, McNeil SE (2009). Nanoparticle interaction with plasma proteins as it relates to particle biodistribution, biocompatibility and therapeutic efficacy. Adv. Drug Deliv. Rev..

[122] Das P (2016). Interaction between a broad-spectrum antibiotic and silver nanoparticles in a human gut ecosystem. J. Nanomed. Nanotechnol..

[123] Di Silvio D, Rigby N, Bajka B, Mackie A, Bombelli FB (2016). Effect of protein corona magnetite nanoparticles derived from bread in vitro digestion on Caco-2 cells morphology and uptake. Int. J. Biochem. Cell Biol..

[124] Lichtenstein D (2015). Impact of food components during in vitro digestion of silver nanoparticles on cellular uptake and cytotoxicity in intestinal cells. Biol. Chem..

[125] Orfi E, Szebeni J (2016). The immune system of the gut and potential adverse effects of oral nanocarriers on its function. Adv. Drug Deliv. Rev..

[126] Choi J (2015). Toxicity of zinc oxide nanoparticles in rats treated by two different routes: single intravenous injection and single oral administration. J. Toxicol. Environ. Health A.

[127] Esmaeillou M, Moharamnejad M, Hsankhani R, Tehrani AA, Maadi H (2013). Toxicity of ZnO nanoparticles in healthy adult mice. Environ. Toxicol. Pharmacol..

[128] Salvia-Trujillo L, Qian C, Martin-Belloso O, McClements DJ (2013). Influence of particle size on lipid digestion and beta-carotene bioaccessibility in emulsions and nanoemulsions. Food Chem..

[129] Shaikh J, Ankola DD, Beniwal V, Singh D, Kumar M (2009). Nanoparticle encapsulation improves oral bioavailability of curcumin by at least 9-fold when compared to curcumin administered with piperine as absorption enhancer. Eur. J. Pharm. Sci..

[130] Sun J (2012). Effect of particle size on solubility, dissolution rate, and oral bioavailability: evaluation using coenzyme Q(10) as naked nanocrystals. Int. J. Nanomed..

[131] Cho HT (2014). Droplet size and composition of nutraceutical nanoemulsions influences bioavailability of long chain fatty acids and Coenzyme Q10. Food Chem..

[132] Walker R, Decker EA, McClements DJ (2015). Development of food-grade nanoemulsions and emulsions for delivery of omega-3 fatty acids: opportunities and obstacles in the food industry. Food Funct..

[133] Katouzian I, Jafari SM (2016). Nano-encapsulation as a promising approach for targeted delivery and controlled release of vitamins. Trends Food Sci. Technol..

[134] Miller ER (2005). Meta-analysis: high-dosage vitamin E supplementation may increase all-cause mortality. Ann. Intern. Med..

[135] Salvia-Trujillo, L., Martin-Belloso, O. & McClements, D. J. Excipient nanoemulsions for improving oral bioavailability of bioactives. *Nanomaterials* **6**, 1–16 (2016). 10.3390/nano6010017 PMC5302540 28344274

[136] Consortium HMP (2012). Structure, function and diversity of the healthy human microbiome. Nature.

[137] Pflughoeft KJ, Versalovic J (2012). Human microbiome in health and disease. Ann. Rev. Pathol..

